# Pectins as Brakes? Their Potential Implication in Adjusting Mesophyll Conductance Under Water Deficit and Salt Stresses

**DOI:** 10.3390/plants14142180

**Published:** 2025-07-14

**Authors:** Margalida Roig-Oliver, Josefina Bota, Jaume Flexas

**Affiliations:** Research Group on Plant Biology Under Mediterranean Conditions, Agro-Environmental and Water Economics Institute (INAGEA), Universitat de les Illes Balears (UIB), Ctra. Valldemossa km 7.5, 07122 Palma, Spain

**Keywords:** abiotic stress, cell wall composition, mesophyll conductance, pectins, photosynthesis, water deficit stress, water use efficiency

## Abstract

Water and salt stresses reduce net CO_2_ assimilation (*A*_N_) primarily by restricting stomatal conductance (*g*_s_) and mesophyll conductance (*g*_m_), while altering leaf structure, anatomy, and cell wall composition. Although some reports observed relationships between these modifications and *g*_m_, in others they remain less clear. Here, we compiled data on studies in which major cell wall components (cellulose; C, hemicellulose; H; pectins; P) were determined with photosynthetic, structural and anatomical features, obtaining a dataset presenting distinct species subjected to both stresses. Among parameters previously reported to affect *g*_m_ (leaf mass per area: *LMA*; chloroplast surface area exposed to intercellular air spaces per unit of leaf surface area: *S*_c_/*S*; fraction of intercellular air spaces: *f*_ias_; cell wall thickness: *T*_cw_), pectins and the P/(C + H) ratio were the unique consistently varying in salt- and water-stressed plants. Despite no single trait correlated with *g*_m_, it was positively linked with [P/(C + H) × *S*_c_/*S* × *f*_ias_]/[*T*_cw_ × Lignin × *LMA*] in studies in which all parameters were tested, suggesting that distinct traits may exert antagonistic influences on *g*_m_. Although further experiments are needed to reinforce our findings, we hypothesize that increases in pectins under stress could limit larger *g*_m_ declines, improving *g*_m_/*g*_s_ ratio and water use efficiency (*WUE*).

## 1. Introduction

Environmental conditions are determinant, influencing plants’ performance and survival. One of the most relevant physiological processes affected by abiotic stresses is net CO_2_ assimilation (*A*_N_). For decades, only stomatal conductance (*g*_s_) and biochemical processes were considered as major *A*_N_ constrainers [1,2,3]. However, nowadays it is widely assumed that mesophyll conductance (*g*_m_)—i.e., the diffusion conductance along the pathway that CO_2_ follows from sub-stomatal cavities to the active carboxylation sites of the Rubisco enzyme at the chloroplast stroma [2]—is another key trait influencing photosynthetic rates either in plants subjected to stresses or along land plants’ phylogeny [3,4,5,6]. Although the mechanistic basis by which *g*_m_ is regulated still requires further investigation [7,8], some authors pointed out that leaf structural traits and anatomical particularities can influence this parameter. In earlier studies, it was assumed that the leaf mass per area (*LMA*) and the leaf density (*LD*) were negatively related to *g*_m_ [9]. Furthermore, sub-cellular anatomical traits such as the chloroplast surface area exposed to intercellular air spaces per unit of leaf surface area (*S*_c_/*S*) and the cell wall thickness (*T*_cw_) strongly influence *g*_m_ [3,6,10,11,12,13,14]. Additionally, some authors also proposed that the fraction of intercellular air spaces (*f*_ias_) could represent another anatomical parameter determining *g*_m_ [14,15,16,17,18]. Besides these relationships between *g*_m_ and foliar/anatomical particularities, some studies have suggested that variations in the chemical composition of the cell wall could also drive *g*_m_ adjustments reviewed in [7,19]. 

The plant cell wall is a complex structure acting as a physical barrier against those stresses encountered during their life. It is primarily compounded by cellulose, hemicellulose, and pectins, but it also contains structural proteins and phenolic compounds such as lignin [19,20,21,22,23]. Among these, cellulose is the most predominant component. It consists of hundreds to over 10,000 (1,4)-β-D-glucose units forming insoluble crystalline microfibrils via hydrogen bonding. Hemicelluloses are enzymatically deposited between cellulose microfibrils, enhancing wall strength. Consequently, this linkage between cellulose and hemicellulose constitutes a robust yet flexible network that prevents cellulose assembling, facilitating wall expansion [22,23,24,25]. This network is embedded within a hydrated pectin matrix compounded by diverse acidic polysaccharides which could influence cell wall porosity, thickness, and flexibility [7,19,21,23,25,26,27,28,29]. Finally, lignin is another key component of the plant cell wall, which deposition may alter its properties [30,31]. Particularly, it is abundant in secondary walls [23,31,32], conferring rigidity, hydrophobicity, and mechanical strength to the cell wall [33]. Even though lignin is primarily composed by aromatic monomers derived from phenylalanine, its precise chemical makeup can vary depending on the developmental stage of the cell, its specific location, and the presence of biotic and/or abiotic stresses [22,31,32,33,34,35]. 

Ellsworth et al. [36] provided the first evidence on the role of changes in cell wall composition affecting *g*_m_. They observed that *Oryza sativa* cell wall mutants with disruptions in cell wall mixed-linkage glucans (MLGs, i.e., diverse polymers belonging to the hemicellulosic cell wall proportion) production significantly decreased *g*_m_ as compared to a wild-type genotype when subjected to high- and low-light intensities. These modifications in cell wall composition were accompanied by variations in anatomical features—specifically, decreased *S*_c_/*S* and *T*_cw_—, which were proposed to have relevant roles driving these *g*_m_ reductions. However, MLGs are specific cell wall compounds of monocots, which, in turn, are known to present particularities regarding their cell wall composition (for instance, low levels of pectins; [37]). From then to now, further experiments have analyzed the implication of variations in cell wall composition—many of them focusing on pectins—influencing *g*_m_ and other parameters. Those studies were addressed testing various species of the same phylogenetic group acclimated to non-stressing conditions [38,39], assessing mutant genotypes acclimated either to favorable [40,41,42] or stressing scenarios [43,44], comparing genotypes of the same species subjected to favorable [45] or stressing conditions [46], or studying one or pairs of species subjected to various treatments [46,47,48,49,50,51,52,53,54,55]. Focusing on the latter experiments—most of them evaluating the effects of distinct water deficit stress regimes—it is remarkable that contrasting results were reported. Although Clemente-Moreno et al. [47] showed a negative relationship between *g*_m_ and the pectins to cellulose plus hemicellulose ratio (i.e., P/(C + H)) in *Nicotiana sylvestris*, *g*_m_ was exclusively linked to modifications in cellulose concentration in *Vitis vinifera* [50]. In *Helianthus annuus* acclimated to short- and long-term water deficit stresses (ST WS and LT WS, respectively) followed by gradual recoveries, *g*_m_ negatively correlated with cell wall bound phenolics, specifically coumaric acid, and lignin [48]. Moreover, this study showed for the first time that variations in the P/(C + H) ratio were linked to modifications in *T*_cw_, the latter being also changes in water use efficiency (*WUE*). Nonetheless, an in-depth analysis of the previous experiment revealed different patterns when evaluating ST WS and LT WS separately since *g*_m_ and the *g*_m_/*g*_s_ ratio were negatively and positively correlated, respectively, with variations in pectins under distinct ST WS levels [51]. However, Luo et al. [16] and Cao et al. [17] proposed that changes in cell wall composition could indirectly affect *g*_m_ by influencing *T*_cw_ in *Brassica napus* and *Lonicera japonica*, respectively, being cellulose a key parameter. Actually, Sun et al. [55] recently observed that water-stressed *Gossypium hirsutum* increased cellulose microfibrils packaging and chelator-soluble pectins content, resulting in enlarged *T*_cw_ and significantly reduced *g*_m_. These results agree with those reported by Hu et al. [44], who tested other *G. hirsutum* genotypes and concluded that cellulose was crucial in driving adjustments in both *T*_cw_ and *g*_m_ under water deprivation. Nonetheless, discrepant findings were also found for the same species, as Yang et al. [53] showed that *g*_m_ was mainly influenced by changes in pectins and in hemicellulose contents under salt stress. In fact, these species-dependent responses could be of higher complexity since they could occur even at genotype level [46]. In that study, two *Solanum lycopersicum* genotypes presenting different strategies to face water scarcity were evaluated under distinct ST WS intensities: a long shelf-life (LSL) genotype, whose fruits remain intact for over 6 to 12 months after harvested, and a non-long shelf-life (nLSL) genotype. On the one hand, the LSL genotype presented significant relationships between *g*_m_ and elastic, sub-cellular anatomical and cell wall compositional adjustments, being the P/(C + H) ratio a key trait. On the other hand, those *g*_m_ adaptations occurring in the nLSL genotype were attributed to changes in foliar structural traits such as *LMA* and to supra-cellular anatomical parameters, particularly, leaf and mesophyll thicknesses (*T*_leaf_ and *T*_mes_, respectively). Finally, when evaluating these *g*_m_ vs. cell wall composition adjustments in pairs of species acclimated to the same stressing environmental conditions, further contrasting findings are observed. Whilst non-significant relationships were detected in *Ginkgo biloba* and *H. annuus* [49], a negative correlation between *g*_m_ and pectins was found in *Hordeum vulgare* and *Triticum aestivum* [52]. Besides this negative relationship between *g*_m_ and pectins, they also positively influenced the *g*_m_/*g*_s_ ratio in both water-stressed *H. annuus* and *S. lycopersicum*, whereas they were only significantly linked with *WUE* in *H. annuus* [55]. Moreover, while pectins and the P/(C + H) ratio correlated positively with *g*_m_ when comparing species belonging to distinct phylogenetic groups acclimated to non-stressing conditions [56], the same relationship is often found to be negative when testing plants under different stresses and/or stress intensity [47,48,55].

We hypothesize that these discrepancies may perhaps be attributed to (i) scale issues, since distinct species exhibited different ranges of *g*_m_ and cell wall compositional traits—in addition to different stress intensities among studies—or (ii) compensatory effects caused by variations under stress of different constrains to *g*_m_ in an antagonistic manner. To test this hypothesis, in this study, we combined data from multiple studies involving distinct species subjected to different degrees of stress. This approach allowed us to broaden the observation scale and to check potential compensatory effects based on antagonistic constraints varying in different directions under stress.

## 2. Results

### 2.1. Relative Effects of Short-Term Water Deficit Stress and Salt Stress on the Studied Parameters

**Figure 1:**
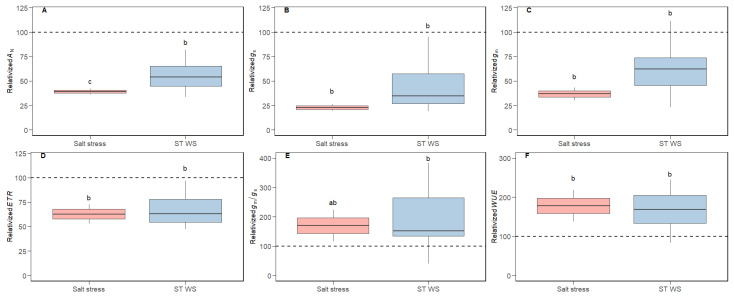
Variability of photosynthetic traits relativized to control (i.e., non-stressing) conditions. Values for plants subjected to salt stress and short-term water deficit stress (“ST WS”) are shown. Dot boxplots represent (**A**) net CO_2_ assimilation (*A*_N_), (**B**) stomatal conductance (*g*_s_), (**C**) mesophyll conductance (*g*_m_), (**D**) electron transport rate (*ETR*), (**E**) *g*_m_ to *g*_s_ ratio (*g*_m_/*g*_s_), and (**F**) intrinsic water use efficiency (i.e., *A*_N_/*g*_s_; *WUE*). The discontinuous horizontal line displays average control values. Different letters indicate significant differences (*p* < 0.05) across experimental conditions according to LSD test, considering that control values are always represented by “a”. Thus, “b” and “c” stand for significant control reductions. Outliers are shown as individual black points.

**Figure 2:**
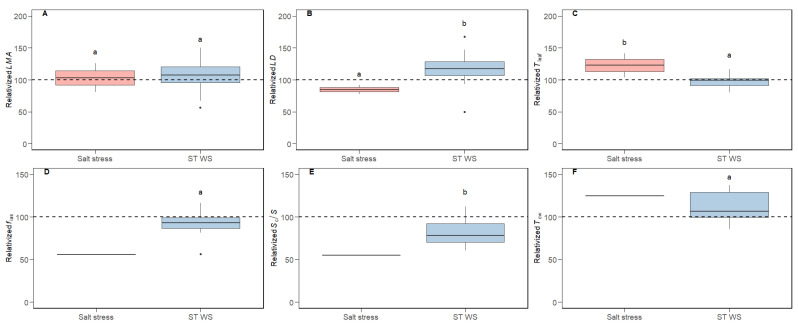
Variability of foliar structural and anatomical traits relativized to control (i.e., non-stressing) conditions. Values for plants acclimated to salt stress and short-term water deficit stress (“ST WS”) are shown. Dot boxplots represent (**A**) leaf mass per area (*LMA*), (**B**) leaf density (*LD*), (**C**) leaf thickness (*T*_leaf_), (**D**) fraction of intercellular air spaces (*f*_ias_), (**E**) chloroplast surface area exposed to intercellular air spaces per unit of leaf surface area (*S*_c_/*S*), and (**F**) cell wall thickness (*T*_cw_). The discontinuous horizontal line displays average control values. Different letters indicate significant differences (*p* < 0.05) across experimental conditions according to LSD test, considering that control values are always represented by “a”. Thus, “b” stand for significant control reductions. Values for *f*_ias_, *S*_c_/*S *and *T*_cw _for salt stress treatment are shown just as a reference and, since *n* = 1 for them, no statistical analysis is presented. Outliers are shown as individual black points.

**Figure 3:**
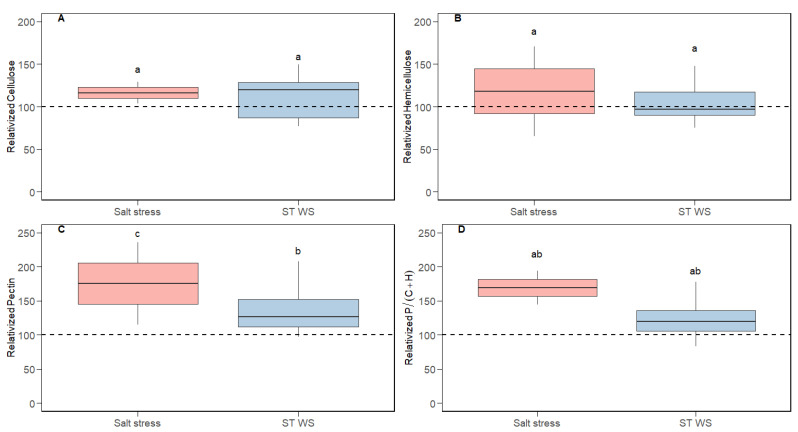
Variability of leaf cell wall composition relativized to control (i.e., non-stressing) conditions. Values for plants acclimated to salt stress and short-term water deficit stress (“ST WS”) are shown. Dot boxplots represent (**A**) cellulose, (**B**) hemicellulose, (**C**) pectin, and (**D**) the pectin to cellulose plus hemicellulose ratio (P/(C + H)). The discontinuous horizontal line displays average control values. Different letters indicate significant differences (*p* < 0.05) across experimental conditions according to LSD test, considering that control values are always represented by “a”. Thus, “b” and “c” stand for significant control reductions.

The variability of photosynthetic traits relativized to CL conditions is shown in Figure 1. Both salt stress and ST WS application resulted in significant alterations in all photosynthetic parameters. For both treatments, the reductions in *A*_N _(Figure 1A) were intermediate between those of *g*_s_, (which were the largest; Figure 1B), those of *g*_m _(Figure 1C) and, especially, *ETR* (which were the lowest; Figure 1D). As a consequence of these relative decreases, the ratios *g*_m_/*g*_s _(Figure 1E) and *A*_N_/*g*_s _(i.e., a proxy for *WUE*; Figure 1F) increased under both stresses. In contrast, non-significant differences across tested conditions were observed for *LMA*, whereas modifications in *LD* and *T*_leaf _were promoted (Figure 2A–C). Also, non-significant effects of short-term water stress were detected for *f*_ias_ and *T*_cw _(Figure 2D,F), while only *S*_c_/*S *displayed significant reductions (Figure 2E). Given that the data we reported for salt stress treatment concerning sub-cellular anatomical parameters only corresponded to a single species (i.e., *n* = 1), no statistical analyses could be addressed. Nonetheless, a tendency may be observed. Thus, salt stress tended to decrease *f*_ias_ and *S*_c_/*S *while increasing *T*_cw _(Figure 2D–F), which was indeed significant in that study when absolute values were compared [36]. Concerning cell wall composition, cellulose and hemicellulose contents were maintained at CL values under salt stress and short-term water deficit stress (Figure 3A,B). However, pectins concentration significantly increased under both stresses (Figure 3C), and so did the P/(C + H) ratio (Figure 3D).

### 2.2. Relationships Between Parameters: Pearson Correlation Matrices in Species Subjected to Salt Stress and Short-Term Water Deficit Stress

The Pearson correlation matrix comprehending absolute values for CL, salt stress, and ST WS is shown in Table 1A. Those relationships with the highest significance (i.e., *p* < 0.01) were mostly detected among the different photosynthetic parameters. Similarly, foliar traits (i.e., *LMA* and *LD*) were highly correlated between them (*R* = 0.69). Even though both parameters—and, especially, *LMA*—were also linked with photosynthetic traits, these correlations were of less significance (i.e., *p* < 0.05). A similar pattern was observed regarding those relationships concerning supra-cellular anatomical parameters, since the correlation between *f*_ias _and *T*_leaf_ was highly significant (*R* = −0.68). Nonetheless, sub-cellular anatomical parameters were linked to distinct photosynthetic traits. In this sense, highly significant relationships between *T*_cw_ and both *WUE* and the *g*_m_/*g*_s_ ratio were found (*R* = 0.64 and 0.75, respectively), whilst *S*_c_/*S* was positively linked with *A*_N _and *g*_s _(*R* = 0.61 in both cases). Concerning cell wall composition, hemicellulose correlated with *A*_N_, *T*_leaf_ and *g*_m_, being only the latter relationship the one with high significance (*R* = −0.54). Finally, pectins were the unique cell wall compound linked to almost all photosynthetic traits, specifically, *A*_N_, *g*_s_, *g*_m_, *ETR*, and *WUE* (*R* = −0.51, −0.48, −0.46, −0.41, and 0.4, respectively).

On the other hand, Table 1B exhibits the Pearson correlation matrix comprehending relativized to CL values for ST WS and salt stress. Clearly, the number of overall correlations diminished in comparison to those reported in Table 1A. Just a few photosynthetic traits still correlated between them, as observed for *A*_N _and *g*_s _(*R* = 0.61) and for the *g*_m_/*g*_s_ ratio and *WUE* (*R* = 0.6), being only the first one of high significance. Interestingly, *LD* was only linked to *LMA* (*R* = 0.88). This relationship was of high significance, as in Table 1A. Moreover, *S*_c_/*S* positively correlated with *LD* (*R* = 0.71). Although pectins were the main cell wall compound in which most significant relationships were detected in Table 1A, here they were not significantly linked with other parameters. However, cellulose negatively correlated with *A*_N_, *g*_m_ and *LD* (*R* = −0.52, −0.57, and −0.55, respectively), whilst hemicellulose was linked with *LMA* and *LD* (both of them presenting *p* < 0.01). Finally, negative relationships between the P/(C + H) ratio and *S*_c_/*S*, *LMA* and *LD* were found (*R* = −0.82, −0.73, and −0.63, respectively), the last ones presenting high significance.

### 2.3. Relationships Among Combined Parameters

**Figure 4:**
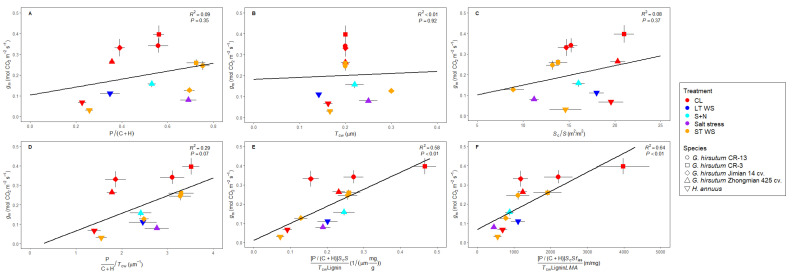
Relationships between mesophyll conductance (*g*_m_) and (**A**) the pectin to cellulose plus hemicellulose ratio (P/(C + H)), (**B**) cell wall thickness (*T*_cw_), and (**C**) chloroplast surface area exposed to intercellular air spaces per unit of leaf surface area (*S*_c_/*S*). Additional relationships between *g*_m_ and combined parameters are shown: (**D**) [P/(C + H)]/*T*_cw_, (**E**) [P/(C + H) × *S*_c_/*S*]/[*T*_cw_ × Lignin], and (**F**) [P/(C + H) × *S*_c_/*S *× *f*_ias_]/[*T*_cw_ × Lignin × *LMA*]. Treatments abbreviations stand for control (“CL”), short- and long-term water deficit stresses (“ST WS” and “LT WS”, respectively), and salt stress plus nanoceria (“S + N”). Data points represent mean absolute values per treatment ± SE, which were compiled from Hu et al. [44], Roig-Oliver et al. [48] and Yang et al. [53].

Three studies reported the complete set of parameters considered here [44,48,53]. As in Table 1A for the pooled dataset, considering absolute mean values for *H. annuus* and *G. hirsutum* subjected to different treatments resulted in non-significant correlations between *g*_m_ and any single parameter (Figure 4A–C). However, when combining parameters, i.e., a ratio of multiplying positive *g*_m_ effectors in the numerator and negative effectors in the denominator, significant relationships emerged. Whilst *g*_m_ and [P/(C + H)]/*T*_cw_ were almost significantly linked (*R*^2^ = 0.29, *p* = 0.07; Figure 4D), a positive and significant relationship between *g*_m_ and [P/(C + H) × *S*_c_/*S*]/[*T*_cw_ × Lignin] emerged (*R*^2^ = 0.58, *p* < 0.01; Figure 4E). This correlation was improved considering *g*_m_ and [P/(C + H) × *S*_c_/*S *× *f*_ias_]/[*T*_cw_ × Lignin × *LMA*] (*R*^2^ = 0.64, *p* < 0.01; Figure 4F).

## 3. Discussion

The average photosynthetic down-regulation under short-term water deficit stress and salt stress we observed in the present compiled small dataset corresponds with that often observed, which nowadays presents a large consensus: huge decreases of *g*_s_ followed by reduced *g*_m_ and much lower *ETR* and biochemistry diminishments, so that photosynthesis is mostly constrained by diffusional limitations under both stresses, except when these become very severe [3,5,57,58,59,60,61]. Despite the large consensus in the sequence and the magnitude of these photosynthetic responses, and in agreement with our dataset, the mechanisms underlying *g*_m_ regulation are still not fully understood [8]. For instance, foliar structure-related parameters are usually impacted by unfavorable environmental conditions [62], but not always [63,64]. In this sense, we only observed significant alterations in *LMA* (Figure 2A–C), although *LD* and *T*_leaf_ were also modified in some of the individual studies included in our dataset [47,48,49,50,51,52,53,65]. Similarly, and concerning the sub-cellular anatomical traits evaluated here (i.e., *f*_ias_,* S*_c_/*S, *and *T*_cw_), some studies have observed significant effects of water shortage on these [14,54,66,67], whilst others have not [68]. In line with this discrepancy, in our dataset we only detected modifications concerning *S*_c_/*S *(Figure 2D–F), but, again, these effects were significant in some experiments we included [44,46,49,53].

Similar disparities have been described regarding changes in cell wall composition. For instance, whilst some authors observed that cellulose was maintained to control values in distinct water-stressed species [48,49,52,55,65,69], others concluded it significantly increased [44,49,50,51,54] or even decreased [43,47,52]. In the same way, discrepancies concerning changes in hemicellulose content have also been observed [44,47,48,49,50,54,65,69]. All this evidence suggests the notion that a particular species—or even cultivars/genotypes of the same species—possess species-dependent cell wall adjustments once subjected to specific stressing conditions. Nonetheless, in most cases, pectins significantly increased due to abiotic stress imposition [44,47,48,49,50,51,52,53,54,55,65,69], as we observed when merging all the compiled data (Figure 3C). Because of these variations in pectins abundance, we also detected significant modifications in their relative proportion (i.e., the P/(C + H) ratio; Figure 3D). Indeed, it has been suggested that pectins could represent a key cell wall component determining cell wall porosity and tortuosity, crucial traits influencing CO_2_ diffusion [7,27,29,70,71]. As they are capable to retain multiple times their own volume in water [27] and that CO_2_ diffuses in solution, Flexas et al. [7] proposed that changes in their content could be accompanied by modifications in their characteristics and in their physicochemical interactions between other wall compounds that could potentially affect the effective porosity to water and CO_2_. In fact, some studies detected that abiotic stresses such as cold or water deficit stress affected pectins’ polymers deposition throughout the leaf mesophyll [42,72,73,74,75], which would finally alter CO_2_ diffusion and, consequently, photosynthesis. Furthermore, these experiments also reported alterations in pectins’ enzymatic performance, representing another fact that would potentially determine *g*_m_. Particularly, Weraduwage et al. [74] tested an *Arabidopsis* mutant genotype and found that the suppression of specific pectin methylesterification enzymes decreased CO_2_ availability and, thus, photosynthesis. More recently, it has been shown that mutant tobacco plants presenting a gene that controls the pectin methylesterification degree significantly decreased *T*_cw_ and increased wall porosity (around 10 and 75% as compared to a wild-type genotype, respectively), which resulted in enlarged *g*_m_ [42]. Therefore, these modifications in pectins’ amounts, physicochemical structure and enzymatic performance could ultimately impact the overall cell wall assembly due to changes in the interactions between all compounds, modifying wall architecture and arrangement. The proposed roles of pectins in improving *g*_m_ have a reflection in their positive correlation when comparing different species across phylogenetic groups [56] and, certainly, they represent the only trait that consistently and significantly increase in response to both water deficit and salt stresses in our dataset (Figure 3).

**Figure 5:**
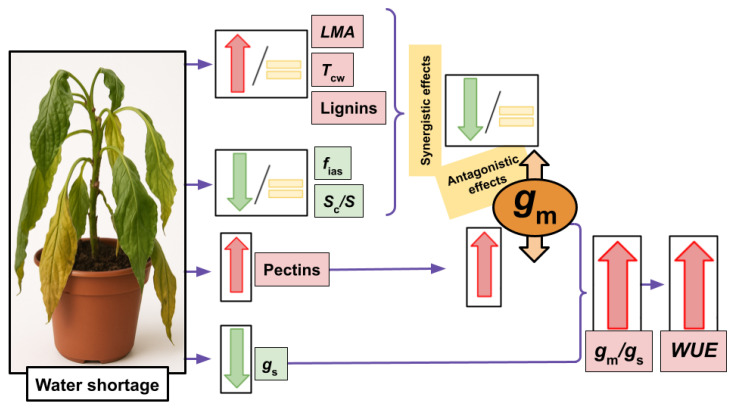
Schematization of the hypothetical mechanism by which *g*_m_ could be regulated under water shortage conditions. The positive effects of increased *f*_ias _and *S*_c_/*S* on *g*_m_ are shown, as well as *g*_m_ constrictions caused by larger *LMA*, *T*_cw_, and increases in both lignin and pectin concentrations. The synergistic effect caused by adjustments in *f*_ias_, *S*_c_/*S*, *LMA*, *T*_cw_ and lignin would provoke maintained or reduced *g*_m_, representing an antagonistic effect to that imposed by higher pectin content. These *g*_m_ adjustments would finally increase the *g*_m_/*g*_s_ ratio and *WUE*. For parameters abbreviations, see Supplementary Table S1.

Besides all this evidence, whilst the relationship between *g*_m_ and pectins and/or the P/(C + H) ratio was evident in some studies performed analyzing distinct abiotic stresses [48,53,54,55], they were not always maintained across tested species, genotypes or experimental conditions [47,49,50,51,52]. Surprisingly, whilst the empirical evidence based on non-stressed species belonging to the same phylogenetic lineage [38] and along land plants’ phylogeny [56] pointed to a positive effect of pectins and P/(C + H) on *g*_m_ (as proposed in Flexas et al. [7]), these correlations under abiotic stress (when appearing) are often negative [47,48,53,54,55]. Thus, we hypothesize that these discrepancies could arise from either scale effects, composite antagonistic effects among different *g*_m_ effectors, or even a combination of both. Although expanding the scale to multiple phylogenetic groups was useful to detect relationships between *g*_m_ and cell wall composition that were not evident in some of the individual phylogenetic lineages [56], expanding the scale does not improve these correlations when focusing on the effects of stress. As shown in Table 1A for those relationships based on absolute values (i.e., expanding the scale by comparing different species), weak relationships were detected between *g*_m_ with *T*_leaf_ and *f*_ias_, whilst they were of high significance with pectins. When repeating these analyses considering water- and salt-stressed values relativized to control (i.e., in an attempt to compare trends rather than expanding the scale), non-significant correlations were found (Table 1B). Therefore, we suggest that the matter of scale is not the main issue for elusive general correlations among *g*_m_ and its previously described effectors when the source of variation is water deficit stress or salt stress. In this sense, and a part from the P/(C + H) ratio, we focused on those parameters which have been widely described as either positive or negative effectors of *g*_m_: *LMA*, *T*_cw_, lignin, *f*_ias_ and *S*_c_/*S*. Regarding the negative ones, it has been shown that larger *LMA* limits the CO_2_ diffusion through the leaf, resulting in decreased *g*_m_ [6,9,76]. Similar effects have been described for thick cell walls (i.e., higher *T*_cw_) [6,18,76,77]. Looking closer at the general trends (Figure 1, Figure 2 and Figure 3), it appears that *LMA* and *T*_cw_ are either kept constant or increased under the tested conditions (Figure 2). As previously explained for main cell wall compounds, lignin content can be increased [52,78], decreased [55,79] or maintained at control values [49,54,55] under abiotic stress imposition. Given that lignin deposition leads to tight molecules packaging, it could influence several cell wall characteristics affecting the CO_2_ supply, such as porosity and tortuosity [30,33]. Consequently, increases in lignin could be viewed as another possible potential negative *g*_m_ effector [49]. On the other hand, and considering positive *g*_m_ effectors, larger *f*_ias_ eases the carbon fixation in chloroplasts stroma [14,15,17]. Finally, *S*_c_/*S* and *g*_m_ have been shown to be positively linked since higher chloroplasts deposition in the leaf mesophyll increases the CO_2_ uptake [6,15,16,17,76,77]. Nonetheless, *f*_ias _and *S*_c_/*S* were either kept constant or decreased in our data (Figure 2). Altogether, we propose that the responses of these five traits under water deficit stress act synergistically to maintain (in the eventual case that all of them are kept at control values) or decrease *g*_m_ (if one or more of them depart from control values in the described directions; see Figure 5). However, pectins and the P/(C + H) ratio consistently increased in response to stress (Figure 3C,D), as reported in the individual studies we considered in our dataset. Therefore, we propose that they could act as positive *g*_m_ effectors, exerting an antagonistic effect to that caused by the five parameters described above that only partially counteracts the overall effects of stress on decreasing *g*_m_. In other words, pectins would be acting as a brake to avoid even larger *g*_m_ declines promoted by the combined effects caused by *LMA*, *T*_cw_, *f*_ias_, *S*_c_/*S*, and lignin. Like “a man pulled by four horses” (in this case, six or more), the direction of the final result would depend on which force is the strongest. However, since most of the “horses” pull in a direction tending to decrease *g*_m_, the final result is a *g*_m_ reduction, but of lower magnitude than it would be without the counteracting effect of pectins. This would explain the negative correlations between pectins and *g*_m_ under abiotic stress despite the described positive effects of the former on *g*_m_. Consequently, the ratio *g*_m_/*g*_s_ would increase due to larger pectins content and, hence, *WUE* [80,81]. The combined synergistic and antagonistic effects we propose are schematized in Figure 5. The likelihood of this hypothesis is supported by the fact that, in a limited dataset considering three studies in which all the tested parameters were analyzed, a significant positive correlation emerges between *g*_m_ and the six combined parameters considering that positive *g*_m_ effectors appear in the numerator and negative ones in the denominator of the equations (Figure 4E,F). Given that we do not have any a priori knowledge on the relative weight of each of these parameters on *g*_m_, we have simply multiplied them without any correction factor, while recognizing that this is a limitation of our approach. However, conceivably this unavoidable limitation results in reduced correlations as compared to an ideal situation in which the relative weight of each factor was known. Consequently, the significant correlations we obtained—despite their acknowledged limitation—are of high value and very promising for future studies in this direction. 

In conclusion, even though we propose a possible mechanism by which pectins could avoid even larger *g*_m_ declines during abiotic stresses imposition, these results must be viewed with care. On the one hand, while here we have used absolute pectin contents—as these were the data available from literature—we agree with the fact that pectin effects on cell wall apparent porosity and tortuosity may strongly depend on factors like, for example, their averaged methylesterification degree, their interaction with C_a_^2+^, etc. (discussed in Flexas et al. [7]). On the other hand, when combining parameters (i.e., multiplying positive effectors in the numerator and negative ones in the denominator of a ratio), we are facing at least two limitations: one is that each parameter has its own units and different ranges of variation, and the second one is that we are inherently assigning each of them the same global weight on influencing *g*_m_, which is probable false but certainly and unknown up to now. However, these parameters combinations do not consider other potential *g*_m_ effectors, for instance, membrane and liquid-phase facilitators such as aquaporins and carbonic anhydrases, respectively [82,83]. Due to these limitations, it is surprising to observe how much the correlation between *g*_m_ and the combined parameters improves in comparison to null relationships among single ones. Therefore, our scarce data-derived hypothesis could establish a new frame on the importance to develop further experiments testing distinct species subjected to other environmental conditions to fully understand *g*_m_ regulation and, thus, photosynthesis and *WUE* regulation under abiotic stresses, which may be used to enhance crops’ productivity [80,81,84].

## 4. Materials and Methods

### 4.1. Data Compilation

We performed a data compilation from published studies in which at least leaf gas exchange and the main cell wall composition parameters were reported in a variety of plant species subjected to either water deficit or salt stress. Within gas exchange parameters, we made sure that *g*_m_ estimations were addressed, since it was the main reference parameter in our study. Concerning main cell wall composition, we focused on cellulose, hemicellulose, and pectins quantifications. To select these reports, we checked that the same procedure for analyzing cell wall composition was conducted. In this sense, the recent paper by Sun et al. [55] was excluded as they used a different methodology for assessing cell wall composition. If foliar structural and anatomical characterization were tested in the experiments, these parameters were also included in the dataset. Thus, we ensured that most of the evaluated traits were quantified in the same plants. The list of the compiled species, the experimental conditions at which they were subjected to, the measured parameters, other relevant information (for instance, genotype), and the original reference are summarized in Supplementary Table S2.

Besides *g*_m_, data for other gas exchange parameters were also extracted from the original studies to be included in the dataset. These parameters were: *A*_N_, *g*_s_, electron transport rate (*ETR*), the *g*_m _to *g*_s _ratio (*g*_m_/*g*_s_), and *WUE* (i.e., *A*_N_/*g*_s_). Cell wall composition features comprised cellulose, hemicellulose, and pectins quantifications. From these values, the P/(C + H) ratio was further calculated. In those experiments in which lignin was additionally quantified, its values were also considered. Foliar structure parameters included *LMA* and *LD*. Finally, anatomical characterization was represented by both supra- and sub-cellular parameters. Whilst the first ones were represented by *T*_leaf_ and *f*_ias_, the second ones included *S*_c_/*S* and *T*_cw_. Compiled data of all the tested parameters in the distinct species can be found in Supplementary Table S3.

Data was classified according to the type of abiotic stress applied to each species. Thus, the experimental conditions were distinguished as: control (“CL”: well-watered plants grown under optimal conditions), short-term water deficit stress (“ST WS”: plants developed under control conditions in which the water supply was reduced and/or stopped in a specific moment of their development), long-term water deficit stress (“LT WS”: plants grown and maintained under water scarcity conditions during their whole development), salt stress (plants irrigated with 150–250 mM NaCl solution at certain moment of their development), and salt stress plus nanoceria (“S + N”: the same conditions applied to the previous treatment in plants in which a polyacrylic acid coated nanoceria was delivered). This classification—specifically, for ST WS, the most analyzed experimental condition across the selected literature and the treatment in which most different stress levels were evaluated—was established regardless of a specific stress intensity applied in a particular experiment. Therefore, we did not focus on stress intensities, but only on types of stresses.

### 4.2. Statistical Analyses

All statistics analyses were made with the R software (ver. 3.2.2; R Core Team, Vienna, 58 Chapter 3 Austria). Since the number of studies that met our requirements was low, we first focused on those treatments presenting a larger number of replicates, being salt stress and ST WS (the number of replicates per parameter and treatment can be found in Supplementary Table S3). Given that the use of absolute values could be species-dependent because they could be influenced by a specific stress or even by different stress intensity, values for all analyzed parameters were relativized to CL to detect trends in response to stress. Then, mean values per species and treatment were used to perform one way ANOVA with subsequent LSD tests to find out statistically significant differences for photosynthetic, foliar structure, cell wall composition, and anatomical parameters across treatments, being significant at *p* < 0.05. After that, Pearson’s correlation matrices were created to find pairwise relationships among all tested parameters using both absolute and relativized values, being considered as significant and highly significant at *p* < 0.05 and *p* < 0.01, respectively. Finally, linear regressions between photosynthetic features, foliar structure, cell wall composition and anatomical characteristics were fitted utilizing mean values per treatment and species. In this case, only the studies that presented data for all the analyzed parameters were tested. Since this resulted in only three selected reports [44,48,53], to increase “*n*” we considered here additional treatments evaluated in these articles: LT WS in *H. annuus* [48] and salt stress plus nanoceria (“S + N”) in *G. hirsutum* [44]. For these analyses, absolute values were compared.

## Figures and Tables

**Table 1:** Pearson correlation matrices between different parameters. Values represent significant (*p* < 0.05) and highly significant (*p* < 0.01) correlation coefficients, respectively. Highly significant correlation coefficients were highlighted in italics, bold and grey color. “Cel.” and “Hemicel.” abbreviations stand for cellulose and hemicellulose, respectively. For other parameters’ abbreviations, see Supplementary Table S1. (**A**) Pearson correlation matrix considering absolute values for control, salt stress and short-term water deficit stress treatments. (**B**) Pearson correlation matrix considering values for salt stress and short-term water deficit stress treatments relativized to control conditions.

(**A**) Significant and highly significant correlations considering absolute values for control, salt stress and short-term water deficit stress treatments.																
	*A*_N_	*g*_s_	*g*_m_	*ETR*	*WUE*	*g*_m_/*g*_s_	*LMA*	*LD*	*T*_leaf_	*f*_ias_	*T*_cw_	*S*_c_/*S*	Cel.	Hemicel.	Pectins	P/(C + H)
*A*_N_		***0.87***	***0.85***	***0.87***	***−0.56***	−0.38	***−0.42***			0.53		***0.61***		−0.35	***−0.51***	
*g*_s_			***0.63***	***0.74***	***−0.78***	***−0.57***	−0.41	−0.38				***0.61***			***−0.48***	
*g*_m_				***0.72***					−0.49	0.53				***−0.54***	***−0.46***	
*ETR*					***−0.45***										−0.41	
*WUE*						***0.81***	0.37				***0.64***				0.4	
*g*_m_/*g*_s_							***0.51***	0.36			***0.75***					
*LMA*								***0.69***		***−0.69***						
*LD*									−0.53							
*T*_leaf_										***−0.68***			0.5	0.43		
*f*_ias_																
*T*_cw_												−0.49				
*S*_c_/*S*																
Cel.																***−0.54***
Hemicel.																***−0.74***
Pectins																
P/(C + H)																
																
(**B**) Significant and highly significant correlations considering relativized to control values for salt and short-term water deficit stresses conditions.																
	*A*_N_	*g*_s_	*g*_m_	*ETR*	*WUE*	*g*_m_/*g*_s_	*LMA*	*LD*	*T*_leaf_	*f*_ias_	*T*_cw_	*S*_c_/*S*	Cel.	Hemicel.	Pectins	P/(C + H)
*A*_N_		***0.61***											−0.52			
*g*_s_																
*g*_m_													−0.57			
*ETR*																
*WUE*						0.6										
*g*_m_/*g*_s_																
*LMA*								***0.88***						***0.82***		***−0.73***
*LD*												0.71	−0.55	***0.77***		***−0.63***
*T*_leaf_																
*f*_ias_																
*T*_cw_																
*S*_c_/*S*																−0.82
Cel.																
Hemicel.																***−0.68***
Pectins																***0.62***
P/(C + H)																

## Data Availability

The data supporting the findings of this study are available from the corresponding author upon request.

## Supplementary Materials

The following supporting information can be downloaded at: https://www.mdpi.com/article/10.3390/plants14142180/s1, Table S1. Number of replicates per parameters and treatments. Experimental conditions were classified as “CL”: non-stressing conditions; “ST WS”: short-term water deficit stress; “LT WS”: long-term water deficit stress; “Salt stress”; “S+N”: salt stress application plus nanoceria delivery, whilst parameters were divided in four categories: gas exchange, leaf structure, cell wall composition, and anatomy. Parameters abbreviations stand for: net CO_2_ assimilation (*A*_N_), stomatal conductance (*g*_s_), mesophyll conductance (*g*_m_), electron transport rate (*ETR*), *g*_m_ to *g*_s_ ratio (*g*_m_/*g*_s_), water use efficiency (*WUE*), the pectins to cellulose plus hemicellulose ratio (P/(C + H)), leaf mass per area (*LMA*), leaf density (*LD*), leaf thickness (*T*_leaf_), fraction of intercellular air spaces (*f*_ias_), chloroplast surface area exposed to intercellular air spaces per unit of leaf surface area (*S*_c_/*S*), and cell wall thickness (*T*_cw_). Table S2. Summary of the evaluated species included in this study. From left to right: species scientific name, particularities (specially relevant information regarding the tested species, such as genotype), experimental conditions (classified as “CL”: non-stressing conditions; “ST WS”: short-term water deficit stress; “LT WS”: long-term water deficit stress; “Salt stress”; “S+N”: salt stress plus nanoceria), measurements addressed in the original reports (divided in four categories: gas exchange, foliar structure, cell wall composition, and anatomy) and references. In the present study, both ST WS and LT WS were used regardless of the specific field capacity percentage evaluated in the original study. For further details, consult the original study. Table S3. Compiled leaf structural, anatomical, cell wall compositional and gas exchange parameters from published studies. “CL”, “ST WS”, “LT WS”, and “S+N” stand for control (i.e., well watering conditions), short-term water deficit stress, long-term water deficit stress, and salt stress application plus nanoceria delivery, respectively. For further details, consult the original study.
