# Supplementary material for: Pectins as Brakes? Their Potential Implication in Adjusting Mesophyll Conductance Under Water Deficit and Salt Stresses
Source: Plants (Basel). 2025 Jul 14;14(14):2180. doi: 10.3390/plants14142180 (PMC12300285; doi:10.3390/plants14142180)
Supplement: Supplementary file 1 [file plants-14-02180-s001.zip › Table S1.pdf]

Table S1. Number of replicates per parameters and treatments. Experimental conditions were classified as "CL": non-stressing conditions; "ST WS": short-term water deficit stress; "LT WS": long-term water deficit stress; "Salt stress"; "S+N": salt stress application plus nanoceria delivery, whilst parameters were divided in four categories: gas exchange, leaf structure, cell wall composition, and anatomy. Parameters abbreviations stand for: net CO_2 assimilation (A_N), stomatal conductance (g_s), mesophyll conductance (g_m), electron transport rate (*ETR*), g_m to g_s ratio (g_m/g_s), water use efficiency (*WUE*), the pectins to cellulose plus hemicellulose ratio (P/(C+H)), leaf mass per area (*LMA*), leaf density (*LD*), leaf thickness (T_{leaf}), fraction of intercellular air spaces (f_{ias}), chloroplast surface area exposed to intercellular air spaces per unit of leaf surface area (S_c/S), and cell wall thickness (T_{cw}).

**Parameters**	**Treatment**				
	**CL**	**Salt stress**	**ST WS**	**LT WS**	**S + N**
**Gas exchange**					
A_N	14	2	12	1	1
g_s	14	2	12	1	1
g_m	14	2	12	1	1
ETR	14	2	12	1	1
g_m/g_s	14	2	12	1	1
WUE	14	2	12	1	1

**Leaf structure**					
LMA	14	2	12	1	1
LD	14	2	11	1	1
**Cell wall composition**					
Cellulose	14	2	12	1	1
Hemicellulose	14	2	12	1	1
Pectins	14	2	12	1	1
P/(C+H)	14	2	12	1	1
Lignin	4	1	2	1	1
**Anatomy**					
T_{leaf}	7	2	6	1	1
f_{ias}	6	1	5	1	1
S_c/S	6	1	5	1	1
T_{cw}	6	1	5	1	1
