# Supplementary material for: Pectins as Brakes? Their Potential Implication in Adjusting Mesophyll Conductance Under Water Deficit and Salt Stresses
Source: Plants (Basel). 2025 Jul 14;14(14):2180. doi: 10.3390/plants14142180 (PMC12300285; doi:10.3390/plants14142180)
Supplement: Supplementary file 1 [file plants-14-02180-s001.zip › Table S2.pdf]

Table S2. Summary of the evaluated species included in this study. From left to right: species scientific name, particularities (specially relevant information regarding the tested species, such as genotype), experimental conditions (classified as "CL": non-stressing conditions; "ST WS": short-term water deficit stress; "LT WS": long-term water deficit stress; "Salt stress"; "S+N": salt stress plus nanoceria), measurements addressed in the original reports (divided in four categories: gas exchange, foliar structure, cell wall composition, and anatomy) and references. In the present study, both ST WS and LT WS were used regardless of the specific field capacity percentage evaluated in the original study. For further details, consult the original study.

Species	Particularities	Experimental conditions	Measurements	References
Nicotiana sylvestris	–	CL	Gas exchange<br>Foliar structure<br>Cell wall composition (excluding lignin)<br>Supra-cellular anatomy	Clemente-Moreno et al. [47]
		Salt stress		
		ST WS		
Arbutus unnedo	Old leaves	CL	Gas exchange<br>Foliar structure<br>Cell wall composition (excluding lignin)	Nadal et al. [65]
		ST WS		
	Young leaves	CL		
		ST WS		
Helianthus annuus	Beauté d'automne cultivar	CL	Gas exchange<br>Foliar structure<br>Cell wall composition<br>Supra- and sub-cellular anatomy	Roig-Oliver et al. [48]
		ST WS		
		LT WS		

Ginkgo biloba	–	CL	Gas exchange<br>Foliar structure<br>Cell wall composition (excluding lignin)<br>Supra- and sub-cellular anatomy	Roig-Oliver et al. [49]
		ST WS		
Helianthus annuus	Beauté d'automne cultivar	CL		
		ST WS		
Vitis vinifera	Grenache cultivar	CL	Gas exchange<br>Foliar structure<br>Cell wall composition (excluding lignin)	Roig-Oliver et al. [50]
		ST WS		
Helianthus annuus	Beauté d'automne cultivar	CL	Gas exchange<br>Foliar structure<br>Cell wall composition	Roig-Oliver et al. [51]
		ST WS		
Triticum aestivum	–	CL	Gas exchange<br>Foliar structure<br>Cell wall composition (excluding lignin)	Roig-Oliver et al. [52]
		ST WS		
Hordeum vulgare	–	CL		
		ST WS		
Solanum lycopersicum	Ailsa Craig non-	CL	Gas exchange	Roig-Oliver et al. [46]

	long shelf-life genotype	ST WS	Foliar structure<br>Cell wall composition (excluding lignin)<br>Supra- and sub-cellular anatomy	
	"de Ramellet" long shelf-life genotype	CL		
		ST WS		
Gossypium hirsutum	Zhongmian 425 cultivar	CL<br>Salt stress<br>S + N	Gas exchange<br>Foliar structure<br>Cell wall composition<br>Supra- and sub-cellular anatomy	Yang et al. [53]
Gossypium hirsutum	Jimian 14 cultivar<br>CR-3 mutant<br>CR-13 mutant	CL	Gas exchange<br>Foliar structure<br>Cell wall composition<br>Supra- and sub-cellular anatomy	Hu et al. [44]
		ST WS		
Helianthus annuus	Beauté d'automne cultivar	CL	Gas exchange<br>Foliar structure<br>Cell wall composition	Roig-Oliver et al. [55]
		ST WS		
Solanum lycopersicum	"de Ramellet" long shelf-life genotype	CL		
		ST WS		
